# Bloom Filter Trie: an alignment-free and reference-free data structure for pan-genome storage

**DOI:** 10.1186/s13015-016-0066-8

**Published:** 2016-04-14

**Authors:** Guillaume Holley, Roland Wittler, Jens Stoye

**Affiliations:** Genome Informatics, Faculty of Technology, Bielefeld University, Bielefeld, Germany; Center for Biotechnology, Bielefeld University, Bielefeld, Germany; International Research Training Group 1906, Bielefeld University, Bielefeld, Germany

**Keywords:** Pan-genome, Similar genomes, Population genomics, Colored de bruijn graph, Bloom filter, Compression, Trie, Index, Succinct data structure

## Abstract

**Background:**

High throughput sequencing technologies have become fast and cheap in the past years. As a result, large-scale projects started to sequence tens to several thousands of genomes per species, producing a high number of sequences sampled from each genome. Such a highly redundant collection of very similar sequences is called a pan-genome. It can be transformed into a set of sequences “colored” by the genomes to which they belong. A colored de Bruijn graph (C-DBG) extracts from the sequences all colored *k*-mers, strings of length *k*, and stores them in vertices.

**Results:**

In this paper, we present an alignment-free, reference-free and incremental data structure for storing a pan-genome as a C-DBG: the bloom filter trie (BFT). The data structure allows to store and compress a set of colored *k*-mers, and also to efficiently traverse the graph. Bloom filter trie was used to index and query different pangenome datasets. Compared to another state-of-the-art data structure, BFT was up to two times faster to build while using about the same amount of main memory. For querying *k*-mers, BFT was about 52–66 times faster while using about 5.5–14.3 times less memory.

**Conclusion:**

We present a novel succinct data structure called the Bloom Filter Trie for indexing a pan-genome as a colored de Bruijn graph. The trie stores *k*-mers and their colors based on a new representation of vertices that compress and index shared substrings. Vertices use basic data structures for lightweight substrings storage as well as Bloom filters for efficient trie and graph traversals. Experimental results prove better performance compared to another state-of-the-art data structure.

**Availability:**

https://www.github.com/GuillaumeHolley/BloomFilterTrie.

## Background

A *stringx* is a sequence of characters drawn from a finite, non-empty set, called the *alphabet*$\mathcal {A}$. Its *length* is denoted by |*x*|. The character at position *i* is denoted by *x*[*i*], the substring starting at position *i* and ending at position *j* by *x*[*i*..*j*]. Strings are concatenated by juxtaposition. If $x = ps$ for (potentially empty) strings *p* and *s*, then *p* is a *prefix* and *s* is a *suffix* of *x*.

A *genome* is the collection of all inheritable material of a cell. Ideally it can be represented as a single string over the DNA alphabet $\mathcal {A} = \{{a},{c},{g},{t}\}$ (or as a few strings in case of species with multiple chromosomes). In practice, however, genomes in databases are often less perfect, either left unchanged in form of the raw data as produced by sequencing machines (millions of short sequences called *reads*), or after some incomplete assembly procedure in form of contiguous chromosome regions (hundreds of *contigs* of various lengths). We are interested in the problem of storing and compressing a set of multiple highly similar genomes, e.g. the pan-genome of a bacterial species, comprising hundreds, or even thousands of strains that share large sequence parts, but differ by individual mutations from one another. An abstract structure that has been proposed for this task is the *colored de Bruijn graph* (C-DBG) [1]. It is a directed graph $G = (V_G,E_G)$ in which each vertex $v \in V_G$ represents a *k*-mer, a string of length *k* over $\mathcal {A}$, associated with a set of colors representing the genomes in which the *k*-mer occurs. A directed edge $e \in E_G$ from vertex *v* to vertex $v'$, respectively from *k*-mer *x* to *k*-mer $x'$, exists if $x[2..k] = x'[1..k-1]$. Each *k*-mer *x* has $|\mathcal {A}|$ possible successors *x*[2..*k*] *c* and $|\mathcal {A}|$ possible predecessors $c x[1..k-1]$ with $c\in \mathcal {A}$. An implementation of such a graph does not have to store edges since they are implicitly given by vertices overlapping on $k-1$ characters.

In this paper, we propose a new data structure for indexing and compressing a pan-genome as a C-DBG, the Bloom Filter Trie (BFT). It is alignment-free, reference-free and incremental, i.e., it does not need to be entirely rebuilt when a new genome is inserted. BFTs provide insertion and look-up operations for strings of fixed length associated with an annotation. This paper is an extended version of the preliminary work presented in [2].

In the next section, existing data structures and software for pan-genome representation are reviewed. The BFT and the operations it supports are then described, followed by the traversal method of a C-DBG stored as a BFT. Finally, experimental results showing the performance of the data structure are provided.

## Existing approaches

The BFT, as well as existing tools for pan-genome storage, uses a variety of basic data structures reviewed in the following. Existing tools for pan-genome storage will then be discussed.

### Data structures

One common way to index and compress a set of strings is the *Burrows-Wheeler Transform* (BWT) [3] that rearranges the input data to enable better compression by aggregating characters with similar context. For multiple sets of strings, a disk-based approach [4] or different terminator characters must be used. The *FM-Index* [5] allows to count and locate the occurrences of a substring in the BWT.

Introduced by Bloom [6], a *Bloom filter* (BF) records the presence of elements in a set. Based on the hash table principle, look-up and insertion times are constant. The BF is composed of a bit array *B*[1..*m*], initialized with 0s, in which the presence of *n* elements is recorded. A set of *f* independent hash functions $h_1, ..., h_f$ is used such that each hash function maps an element to an integer from one to *m*. Inserting an element *e* into *B* and testing for its presence are then

$$\begin{aligned} \textsf {Insert}(e,B): B[h_i(e)] \leftarrow 1 \quad \text { for all } i = 1,...,f \end{aligned}$$

and

$$\begin{aligned} \textsf {MayContain}(e,B) : \bigwedge \limits _{i=1}^{f}B[h_i(e)], \end{aligned}$$

respectively, where $\bigwedge$ is the logical conjunction operator. The BF does not generate false negatives but may generate false positives, as $\textsf {MayContain}$ can report the presence of elements which are not present but a result of independent insertions.

The *Sequence Bloom Tree* (SBT) [7] is a binary tree with BFs as vertices. An internal vertex is the union of its two children BFs, i.e., a BF where a slot is set to 1 if the slot at the same position in at least one of the two children is 1.

A *trie* [8] is a rooted edge-labeled tree $T = (V_T,E_T)$ storing a set of strings. Each edge $e \in E_T$ is labeled with a character and no two edges starting at the same vertex can have the same character. A path from the root to a leaf represents the string obtained by concatenating all the characters on this path. The depth of a vertex *v* in *T* is denoted by *depth*(*v*, *T*) and is the number of edges between the root of *T* and *v*. The height of *T*, denoted by *height*(*T*), is the number of edges on the longest path from the root of *T* to a leaf. The *burst trie* [9] is an efficient implementation of a trie which reduces its number of branches by compressing sub-tries into leaves. Its internal vertices are labeled with multiple prefixes of length 1, linked to children. The leaves are labeled with multiple suffixes of arbitrary length. A leaf has a limited capacity of suffixes and is *burst* when this capacity is exceeded. A burst splits suffixes of a leaf into prefixes of length 1, linked to new leaves representing the remaining suffixes.

### Software for pan-genome storage

Existing tools for pan-genome storage are mostly alignment-based or reference-based and take a set of assembled genomes as input. Alignments naturally exhibit shared and unique regions of the pan-genome but are computationally expensive to obtain. In addition, misalignments can lead to an inaccurate estimation of the pan-genome regions [10]. PanCake [11] is an extension of string graphs, known from genome assembly [12], which achieves compression based on pairwise alignments. Experiments showed compression ratios of 3:1 to 5:1. Nguyen et al. [13] formulated the pan-genome construction problem as an optimization problem of arranging alignment blocks for a set of genomes partitioned by homology. The complexity of the problem has been shown to be NP-hard, and a heuristic using Cactus graphs [14] was provided. However, a multiple sequence alignment is required for creating the blocks, another NP-hard problem.

Among the reference-based tools, Huang et al. [15] proposed to build a pan-genome by annotating a reference genome with all the variants detected between a set of genomes and the reference. The BWT of the augmented reference is then computed and can be used by an aligner based on the FM-Index. While being more accurate with the augmented reference genome than BWA [16] with the reference alone, the aligner is between 10 to 100 times slower, uses significantly more memory and can introduce false positive alignments. RCSI [17] (Referentially Compressed Search Index) uses referential compression with a compressed suffix tree to store a pan-genome and to search for exact or inexact matches. The inexact matching allows a limited number of edit distance operations. 1092 human genomes totaling 3.09 TB of data were compressed into an index of 115 GB, offering a compression ratio of about 28:1. Yet, the index is built for a maximum length query and a maximum number of edit operations. MRCSI [18] improves on RCSI by proposing a compressed search index based on multiple references.

Closer to our approach is SplitMEM [19], which uses a C-DBG to build a pan-genome from assembled genomes and extract the shared regions. The C-DBG is directly constructed in a compressed way, where a non-branching path is stored in a single vertex, using an augmented suffix tree. Baier et al. [20] improved SplitMEM in theory and practice with two algorithms that use the BWT and a compressed suffix tree. Unfortunately, both tools use more memory than the original size of the input sequences.

The tool Khmer [21] provides a lightweight representation of de Bruijn graphs [22] based on Bloom filters and a graph labeling method based on graph partitioning. Unfortunately, the graph labeling method does not offer yet enough flexibility to reproduce the experiments presented in this paper.

The SBT data structure has been implemented in an alignment-free, reference-free and incremental software [7] to label raw sequences with their colors. The proposed tool is designed to index and compress data from sequencing experiments for effective query of full-length genes or transcripts by separation into *k*-mers. A leaf of an SBT is used to represent a sequencing experiment by extracting all its *k*-mers and storing them in the BF of the leaf. The SBT software does not represent exactly the set of *k*-mers of the sequencing experiments they contain, though, due to the inexact nature of BFs.

## The Bloom Filter Trie

The Bloom Filter Trie (BFT) that we propose in this paper is an implementation of a C-DBG. It is based on a burst trie and is used to store *k*-mers associated with a set of colors. For the moment we may assume that colors are represented by a bit array *color* initialized with 0s. Each color has an index $i_{color}$ such that ${color}_x[i_{color}] = 1$ records that *k*-mer *x* has color $i_{color}$. Sets of colors will later be compressed. All arrays in a BFT are dynamic: An insertion at position *pos* in an array *A* reallocates it and shifts every slot having an index $\ge {pos}$ by one position in $\mathcal {O}(|A|)$ time.

In the following, let $t = (V_t,E_t)$ be a BFT created for a certain value of *k* where we assume that *k* is a multiple of an integer *l* such that *k*-mers can be split into $\frac{k}{l}$ equal-length substrings. The maximum height of *t* is ${height}_{max}(t) = \frac{k}{l}-1$. The alphabet we consider is the DNA alphabet $\mathcal {A} = \{\textit{a},\textit{c},\textit{g},\textit{t}\}$, and because $|\mathcal {A}| = 4$, each character can be stored using two bits. A vertex in a BFT is a list of containers, zero or more of which are *compressed*, plus zero or one *uncompressed* container. In the following, we will explain how the containers are represented and how an uncompressed container is burst when its capacity is exceeded.

### Uncompressed container

**Fig. 1:**
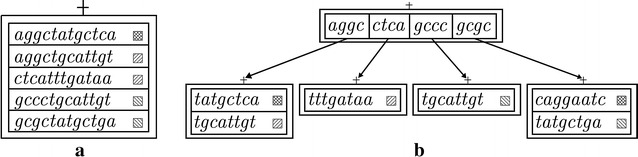
Insertion of six suffixes (that are here complete *k*-mers) with different colors (*boxes* with *diagonal lines*) into a BFT with $k=12$, $l=4$ and $c=5$. In **a**, the first five suffixes are inserted at the root into an uncompressed container. When a sixth suffix *gcgccaggaatc* is inserted, the uncompressed container exceeds its capacity and is burst, resulting in the BFT structure shown in **b**

An uncompressed container of a vertex *v* in a BFT is a limited capacity set of tuples ${<}s,{color}_{ps}{>}$ where *s* is a suffix and *p* is the prefix represented by the path from the root to *v* such that $|p|+|s| = k$. Tuples are lexicographically ordered in the set according to their suffixes. Uncompressed containers are burst when the number of suffixes stored exceeds their capacity $c > 0$. Then, each suffix *s* of the uncompressed container is split into a prefix $s_{pref}$ of length *l* and a suffix $s_{suf}$ of length $|s|-l$ such that $s = s_{pref}s_{suf}$. Prefixes are stored in a new compressed container. Suffixes, attached with their colors, are stored in new uncompressed containers, themselves stored in the children of the compressed container. An example of a BFT and a bursting is given in Fig. 1.

### Compressed container

A bursting replaces an uncompressed container by a compressed one, used to:

- store $q \le c$ suffix prefixes in compressed form (in Fig. 1b, $q=4$),
- store links to children containing the suffixes, and
- reconstruct suffix prefixes and find the corresponding children.

To store a suffix prefix $s_{pref}$ efficiently, it is split into a prefix *a* and a suffix *b* with respective binary representations $\alpha$ and $\beta$ of length $\lambda$ and $\mu$ bits. A compressed container is composed of four structures *quer*, *pref*, *suf* and *clust*, where:

- *quer* is a BF represented as a bit array of length *m* and *f* hash functions, used to record and filter for the presence of *q* suffix prefixes;
- *pref* is a bit array of $2^{\lambda }$ bits initialized with 0s and used to record prefix presence exactly. Here the binary representation $\alpha$ of a prefix *a* is interpreted as an integer such that ${pref}[\alpha ]$ set to 1 records the presence of *a*;
- *suf* is an array of *q* suffixes *b* sorted in ascending lexicographic order of the original suffix prefixes they belong to;
- *clust* is an array of *q* bits, one per suffix of array *suf*, that represents cluster starting points. A cluster is a list of consecutive suffixes in array *suf* that share the same prefix. It has an index $i_{cluster}$ with $1 \le i_{cluster} \le 2^{\lambda }$ and a start position ${pos}_{cluster}$ in the array *suf* with $i_{cluster} \le {pos}_{cluster} \le q$. Position *pos* in array *clust* is set to 1 to indicate that the suffix in *suf*[*pos*] starts a cluster because it is the lexicographically smallest suffix of its cluster. A cluster contains $n \ge 1$ suffixes and, therefore, position *i* in array *clust* is set to 0 for ${pos} < i < {pos}+n$. The end of a cluster is indicated by the beginning of the next cluster or if ${pos} \ge q$.

For example, the internal representation of the compressed container shown in Fig. 1b with $|a|=2$ and $|b|=2$ would be:
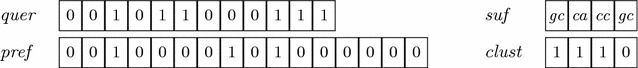


The size of *q* substrings in a compressed container is $m + 2^{\lambda } + q \cdot (\mu + 1)$ bits. A bursting minimizes this size by choosing a prefix length |*a*| and a BF size *m* such that the set of substrings stored in a compressed container does not occupy more memory than their original representation in an uncompressed container, i.e., $m + 2^{\lambda } \le q \cdot (\lambda - 1)$. Each suffix prefix inserted after a bursting costs only $\mu + 1$ bits. When the average size per suffix prefix stored is close to $\mu + 1$ bits, arrays *pref*, *suf* and *clust* can be recomputed by increasing |*a*| and decreasing |*b*|, such that $2^{\lambda '} + q \cdot \mu ' < 2^{\lambda } + q \cdot \mu$, where $\lambda '$ and $\mu '$ are the values of $\lambda$ and $\mu$, respectively, after resizing.

## Operations supported by the Bloom Filter Trie

The BFT supports all operations necessary for storing, traversing and searching a pan-genome, as well as to extract the relevant information of the contained genomes and subsets thereof. Here we describe the most basic ones of them, Look-up and Insertion, as well as how the sets of colors are compressed. The traversal of the graph is discussed in the next section.

The algorithms use three auxiliary functions. $\textsf {HammingWeight}(\alpha ,{pref})$ counts the number of 1s in ${pref}[1..\alpha ]$ and corresponds to how many prefixes represented in array *pref* are lexicographically smaller than or equal to an inserted prefix *a* with binary representation $\alpha$ of length $\lambda$ bits. This requires $\mathcal {O}(2^{\lambda })$ time. The second function, $\textsf {Rank}(i,{clust})$, iterates over array *clust* from its first position until the *i*th entry 1 is found and returns the position of this entry. It corresponds to the start position of cluster *i* in array *clust*. If the entry is not found, the function returns $|{clust}|+1$ as a position. While $\textsf {Rank}$ could be implemented in $\mathcal {O}(1)$ time [5], we use a more naive but space efficient $\mathcal {O}(q)$ time implementation, where *q* is the number of suffix prefixes in a compressed container. Finally, $\textsf {BinarySearch}({uc},{s})$ searches for the suffix *s* in the uncompressed container *uc* in $\mathcal {O}(\log _2 c)$ time, where *c* is the capacity of *uc*.

### Look-up

The function that tests whether a suffix prefix $s_{pref} = ab$ with binary representation $\alpha \beta$ is stored in a compressed container *cc* is given in Algorithm 1. Line 1 verifies the presence of prefix *a* in the array *pref* in $\mathcal {O}(1)$ time. If *a* is present, line 2 computes in $\mathcal {O}(2^{\lambda })$ time the Hamming weight *i* of *a*, i.e., the index of the cluster in which suffix *b* is possibly situated. Line 3 locates the rank of *i*, i.e., the start position of the cluster, and lines 4–7 compare the suffixes of the cluster to *b*. Lines 3–7 are computed in $\mathcal {O}(q)$ time. Algorithm 1 has therefore a worst case running time of $\mathcal {O}(2^{\lambda } + q)$.

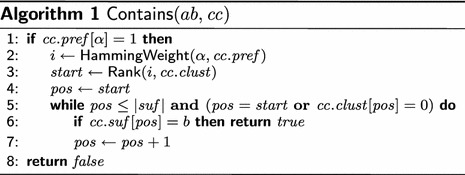



The function that tests whether a *k*-mer *x* is present in a BFT $t = (V_t,E_t)$ is given in Algorithm 2. Each vertex $v \in V_t$ represents *k*-mer suffixes possibly stored in its uncompressed container or rooted from its compressed containers. The look-up traverses *t* from the root and, for a vertex *v*, queries its containers one after the other for suffix $x_{suf} = {x[l \cdot {depth}(v,t) + 1..k]}$. If the queried container is compressed, its BF *quer* is queried for $x_{suf}[1..l]$ using the function $\textsf {MayContain}$ in $\mathcal {O}(f)$ time where, as above, *f* is the number of hash functions used by the BF. In case of a positive answer, the function $\textsf {Contains}$ is used for an exact membership of $x_{suf}[1..l]$. If it is found, the traversing procedure continues recursively on the corresponding child. The absence of $x_{suf}[1..l]$ indicates the absence of *x* in *t* since $x_{suf}[1..l]$ cannot be in another container of *v* because of the insertion process explained later in this paper. If the container is uncompressed, the presence of $x_{suf}$ is detected using the function $\textsf {BinarySearch}$. As an uncompressed container has no children, a match indicates the presence of the *k*-mer. Algorithm 2 is initially called as $\textsf {TreeContains}(x, 1, l, {root})$. In the worst case, all vertices on a traversed path represent all possible suffix prefixes and the BFs *quer* have a false positive ratio of 0. In such case, each traversed vertex contains $\left\lceil \frac{|\mathcal {A}|^l}{c}\right\rceil$ containers. The longest path of a BFT has $\frac{k}{l}$ vertices. Therefore, the worst case time of $\textsf {TreeContains}$ is $\mathcal {O}\left (\frac{k}{l} \cdot \left (\left\lceil \frac{|\mathcal {A}|^l}{c}\right\rceil \cdot f + 2^{\lambda } + q\right )\right)$.

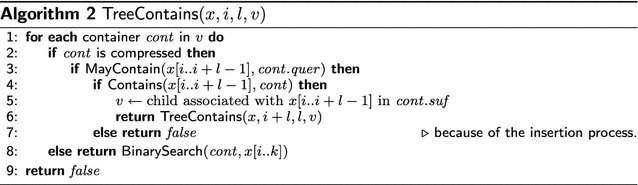



### Insertion

Prior to any *k*-mer insertion into a BFT *t*, a look-up verifies if the *k*-mer is already present. If it is, only its set of colors is modified. Otherwise, the look-up stops the trie traversal on a container *cont* of a vertex *v* where the searched suffix prefix or *k*-mer suffix is not present. If *cont* is uncompressed, the insertion of the *k*-mer suffix and its color is a simple $\mathcal {O}(\log _2 c)$ time process. If *cont* is compressed, the insertion of suffix prefix $s_{pref} = ab$ is a bit more intricate. In fact, it will only be triggered if *cont* is the first compressed container of *v* to have $s_{pref}$ as a false positive ($\textsf {MayContain}(s_{pref},{cont}.{quer}) = {true}$ and $\textsf {Contains}(s_{pref},{cont}) = {false}$). False positives are therefore “recycled”, which is a nice property of BFTs: The BF *quer* remains unchanged, and only *pref*, *suf* and *clust* need to be updated in a way similar to Algorithm 1. The presence of prefix *a* must be first verified by testing the value of ${pref}[\alpha ]$ where $\alpha$ is the binary representation of *a*. If ${pref}[\alpha ] = 0$, prefix *a* is not present and is recorded by setting ${pref}[\alpha ]$ to 1. Then, the index ${id}_{cluster}$ and start position ${pos}_{cluster}$ of the new cluster are computed using $\textsf {HammingWeight}$ and $\textsf {Rank}$. The suffix *b* is inserted into ${suf}[{pos}_{cluster}]$ and a 1 into ${clust}[{pos}_{cluster}]$. This takes $\mathcal {O}(2^{\lambda } + 2q)$ time. If ${pref}[\alpha ] = 1$ prior to insertion, prefix *a* is already present, and ${id}_{cluster}$ and ${pos}_{cluster}$ have already been computed by $\textsf {Contains}(s_{pref},{cont})$. Let *n* be the number of suffixes in cluster ${id}_{cluster}$. Suffix *b* is inserted into *suf*[*pos*] such that ${pos}_{cluster} \le {pos} \le {{pos}_{cluster}+n}$ and ${suf}[{pos}-1] < {suf}[{pos}]$. If ${pos} = {pos}_{cluster}$, *b* starts its cluster: A 1 is inserted into *clust*[*pos*] and ${clust}[{pos}+1]$ is set to 0. Otherwise, a 0 is inserted into *clust*[*pos*]. This takes $\mathcal {O}(2q)$ time. The worst case insertion time of a *k*-mer is $\mathcal {O}(d + 2^{\lambda } + 2q)$ with *d* being the worst case time look-up.

The internal representation of the compressed container shown in Fig. 1b after insertion of the suffix prefix *gtat* is given below (inserted parts are highlighted). The presence of prefix *gt* is recorded in *pref*[12]. Then, its cluster index and start position are computed as 4 and 5, respectively. Consequently, after reallocation of arrays *suf* and *clust*, suffix *at* is inserted in *suf*[5] and *clust*[5] is set to 1 to indicate that *suf*[5] starts a new cluster.
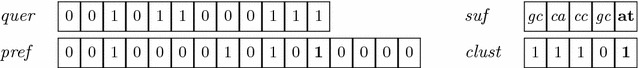


### Color compression

Remember from the BFT description that color sets associated with *k*-mers in a C-DBG are initially stored as bit arrays in BFTs. However, these can be compressed by storing sets of colors that are identical for multiple *k*-mers once. To this end, a list of all color sets occurring in the BFT is built and sorted in decreasing order of total size, i.e., the number of *k*-mers sharing a color set multiplied by its size. Then, by iterating over the list, each color set is added incrementally to an external array if the integer encoding its position in the array uses less space than the size of the color set itself. Finally, each color set present in the external array is replaced in the BFT by its position in the external array.

## Traversing successors and predecessors

Let *t* be a BFT that represents a C-DBG *G*. For a *k*-mer *x*, visiting all its predecessors or successors in *G*, denoted *pred*(*x*, *G*) and *succ*(*x*, *G*), respectively, implies the look-up of $|\mathcal {A}|$ different *k*-mers in *t*. Such a look-up would visit in the worst case $|\mathcal {A}| \cdot ({height}_{max}(t)+1)$ vertices in *t*. This section describes how to reduce the number of vertices and containers visited in *t* during the traversal of a vertex in *G*.

### **Observation 1**

Let *G* be a C-DBG represented by a BFT *t* and *x* a *k*-mer corresponding to a vertex of *G*. All *k*-mers of *succ*(*x*, *G*) share *x*[2..*k*] as a common prefix and therefore share a common subpath in *t* starting at the root. On the other hand, *k*-mers of *pred*(*x*, *G*) have different first characters and, therefore, except for the root of *t* do not share a common subpath. Hence, the maximum number of visited vertices in *t* for all *k*-mers of *succ*(*x*, *G*) is $1 + {height}_{max}(t)$ and for all *k*-mers of *pred*(*x*, *G*) is $1 + |\mathcal {A}| \cdot {height}_{max}(t)$.

### **Lemma 1**

Let *G* be a C-DBG represented by a BFT *t*, *x* a *k*-mer in *t* and *v* a vertex of *t* that terminates the shared subpath of the *k*-mers in *succ*(*x*, *G*). If ${depth}(v,t) = {height}_{max}(t)$, *succ*(*x*, *t*) suffixes may be stored in any container of *v*. If not, they are stored in the uncompressed container of *v*.

### *Proof*

A vertex *v* is the root of a sub-trie storing *k*-mer suffixes of length ${l \cdot ({height}_{max}(t) - {depth}(v,t) + 1)}$ with $l = \frac{k}{{height}_{max}(t)+1}$. Let *s* be a *k*-mer suffix of *succ*(*x*, *t*) rooted at a vertex $v \in V_t$. If ${depth}(v,t) \ne {height}_{max}(t)$ but *s* is rooted at a compressed container in *v*, then this compressed container stores *s*[1..*l*], and $s[l+1..|s|]$ is rooted in one of its children. As the divergent character between the *k*-mer suffixes of *succ*(*x*) is in position $|s| - 1$, this character is in $s[l+1..|s|]$, rooted at one child of this compressed container. Therefore *v* does not terminate the common subpath shared by *succ*(*x*, *t*) *k*-mers. $\square$

Lemma 1 proves that the only two cases where a look-up of *pred*(*x*, *G*) or *succ*(*x*, *G*) must search in different containers of a vertex are:

- searching at the root of *t* for *k*-mers of *pred*(*x*, *G*),
- if ${depth}(v,t) = {height}_{max}(t)$, searching at vertex *v* for suffixes of *succ*(*x*, *G*).

Restricting the hash functions used in the compressed containers to take only positions 2 through $l-1$ into account, allows to limit the search space.

### **Lemma 2**

Let *t* be a BFT where the *f* hash functions $h_i$ of *quer* have the form $h_i(s_{pref}):s_{pref}[2..l-1]\rightarrow \{1,..,m\}$ for $i=1,...,f$. Then, for a vertex *v* of *t* and a suffix prefix $s_{pref}$, all possible substrings $s'_{pref} = c_1 s_{pref}[2..l-1] c_2$ are contained in the same container of *v*.

### *Proof*

Assume a *k*-mer suffix *s* inserted in a vertex *v* of *t*. A look-up for *s* analyzes the containers of *v* from the head to the tail of the container list. In the worst case, *s* can be rooted, according to BFs *quer*, in all compressed containers as a true positive or as a false positive. However, a look-up stops either on the first compressed container claiming to contain the suffix prefix $s_{pref} = s[1..l]$, or on the uncompressed container. As the hash functions of *quer* consider only $s_{pref}[2..l-1]$, a look-up will therefore stop on the same container for any substring $s'_{pref} = c_1 s_{pref}[2..l-1] c_2$. $\square$

As a consequence of Lemma 2, each suffix prefix $s_{pref}$ stored or to store in arrays *pref*, *suf* and *clust* is modified such that $s'_{pref} = s_{pref}[2..l] s_{pref}[1]$, which guarantees that all $s''_{pref} = s'_{pref}[1..l-2] c_2 c_1$ are in the same container. Furthermore, suffixes stored in array *suf* are required to have a minimum length of two characters to ensure that characters $c_1$ and $c_2$, the variable parts between the different $s''_{pref}$, are stored in array *suf*. Hence, as all $s''_{pref}$ share $s'_{pref}[1..l-2]$ as a prefix, they share the same cluster in arrays *suf* and *clust*. Suffix prefixes $s''_{pref} = s'_{pref}[1..l-1] c_1$ also have consecutive suffixes in their cluser.

## Evaluation

We compared BFT, version 0.5, to SBT [7], version 0.3.5, on a mid-class laptop with an SSD hard drive and an Intel Core i5-4300M processor cadenced at 2.6 GHz, using a single thread. It was not possible to include Khmer in this evaluation as it does not support $k > 32$ and building a labeled de Bruijn graph with it requires concatenated raw sequence files as input, where it is not possible to specify a minimum number of occurences per *k*-mer. Results provided in this section can differ from those reported in the preliminary version of this paper [2] as evaluated software versions are different and computational cost of *k*-mer counting is now excluded. Also main memory usage is now provided in addition to the disk space usage. BFT and SBT were used to represent one real and one simulated pan-genome dataset. The real dataset consists of raw sequencing data from 473 clinical isolates of *Pseudomonas aeruginosa* sampled from 34 patients (NCBI BioProject PRJEB5438), resulting in 820.76 GB of FASTQ files. The simulated dataset corresponds to 154 isolates generated from 19 strains of *Yersinia pestis*. For each isolate, we used Wgsim [23] to create 6,000,000 reads of length 100 with a substitution sequencing error rate of 0.5 %, resulting in 233.84 GB of FASTQ files. We first used KmerGenie [24] on a subsample of the files for each dataset to estimate the best *k*-mer length and the minimum number of occurrences for considering a *k*-mer valid (not resulting from a sequencing error). A length of $k=63$ with a minimum number of 3 occurrences was selected for the real and a length of $k=54$ with a minimum of 15 occurrences for the simulated data set. The capacity *c* influences the compression ratio as well as the time for insertion and look-up. We chose a value of $c=248$, as it showed a good compromise in practice. The prefix length *l* determines the size of several internal structures of the BFT and how efficiently they can be stored. We selected $l=9$, as this limits the internal fragmentation of the memory. As the size of BFs used by the SBT software must be specified prior to the *k*-mer insertion and should be the same for all vertices, the authors of SBT suggested to estimate the number of unique *k*-mers in each dataset to design the size of BFs, at the price of an extra computation time (personal communication). Since we knew the exact number of unique *k*-mers from the BFT construction, we used this instead: 93,201,551 63-mers for the real dataset and 37,334,323 54-mers for the simulated dataset, resulting in BF sizes of 11.11 MB and 4.45 MB, respectively. We also reused unique *k*-mer counts computed by the BFT to estimate the number of hash functions to use in SBT: One hash function for the real dataset and two hash functions for the simulated dataset. Insertion time and memory usage are shown in Table 1. Insertion time and peaks of memory include the compression steps proposed by both tools, i.e., color compression and RRR compression [25], respectively. The SBT disk sizes are given for the leaves first, since the internal vertices can be reconstructed from them, and then for the complete tree. The compressed disk size corresponds to the size of both data structures on disk, compressed using 7z [26] with the highest compression level and LZMA2 [26] as compression method.

**Table 1:** Insertion time and memory usage for the real (*P. aeruginosa*) and simulated (*Y. pestis*) dataset. The compression ratio is given w.r.t. the original file sizes. Disk sizes for the SBT are given for the leaves first and then for the complete tree

	*P. aeruginosa*		*Y. pestis*	
	BFT	SBT	BFT	SBT
Insertion time (min)	168.52	371.45	29.88	32.67
Peak of main memory (MB/compr. ratio)	7487/112:1	7356/114:1	1313/182:1	1586/151:1
Disk size (MB/compr. ratio)	1644/511:1	2076–4572/405:1–184:1	484/495:1	538–1117/445:1–214:1
Compressed disk size (MB/compr. ratio)	833/1009:1	1906–4280/441:1–196:1	225/1064:1	528–1099/454:1–218:1

**Fig. 2:**
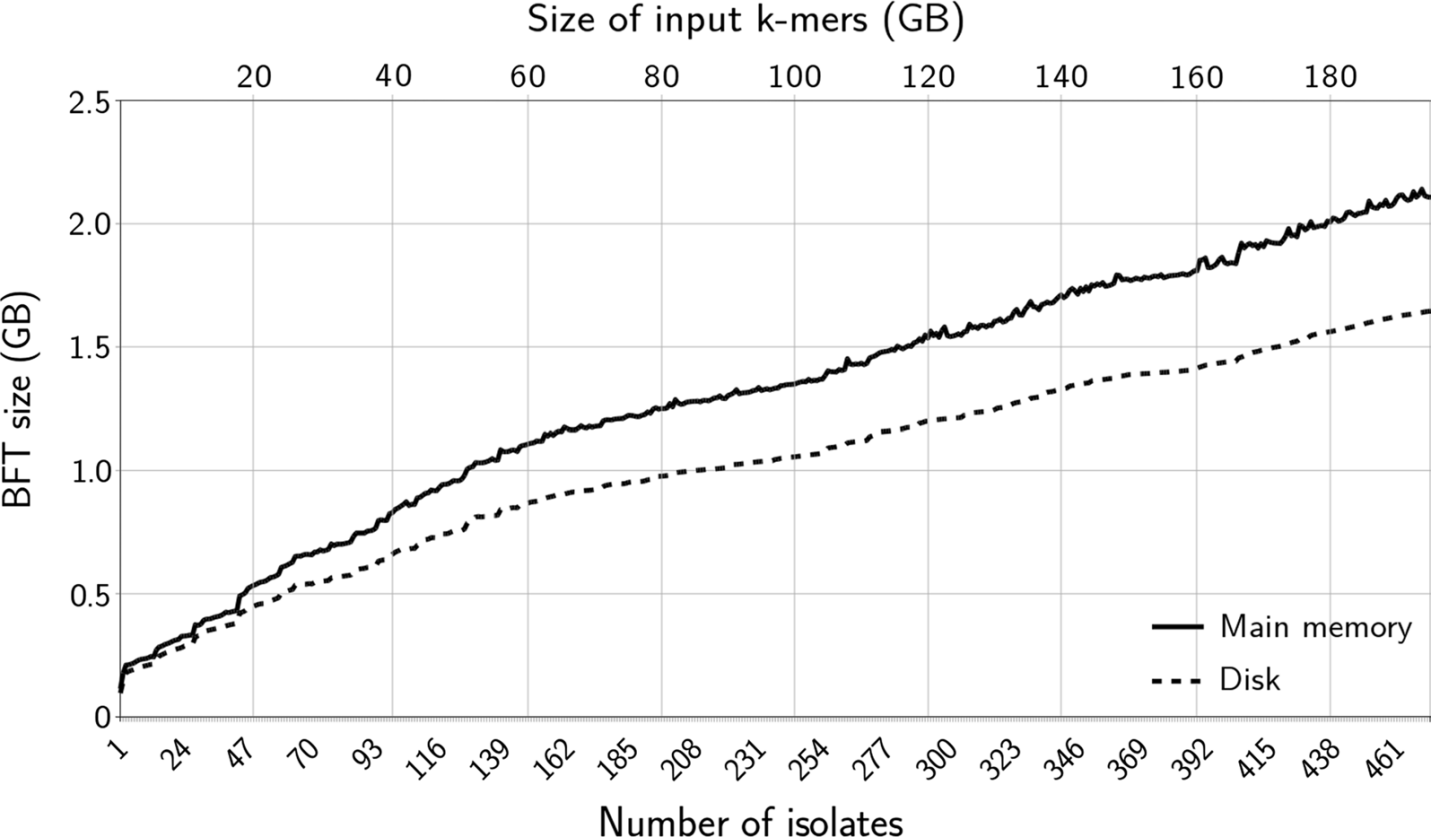
BFT main memory and disk size for pan-genomes made of one up to all *P. aeruginosa* isolates

**Fig. 3:**
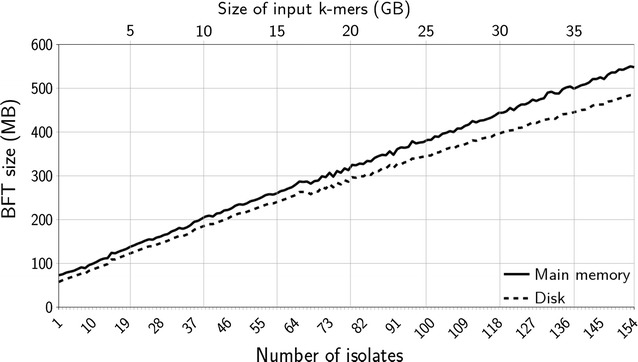
BFT main memory and disk size for pan-genomes made of one up to all simulated *Y. pestis* isolates

We suspect that 7z delivers such a high compression ratio for the BFT because it takes advantage of the data redundancy among the uncompressed containers, particularly among the sets of colors. Indeed, the color compression step used by the BFT is rather simple but keeps the sets of colors fully indexed and, therefore, does not penalize insertion time. In contrast, SBT uses a more advanced compression method, RRR, which explains the lower compression ratio offered by 7z. The final size of the BFT in main memory and on disk for all pan-genomes made of one up to all isolates for the real and simulated dataset is shown in Figs. 2 and 3, respectively. As shown, the memory growth of the BFT is largely sub-linear with respect to the size of the input data.

For each dataset, a set of randomly selected *k*-mers of the BFT was written to disk and reused as a batch query for both data structures. Real and simulated dataset batch queries contain ten million 63-mers and 54-mers, respectively. Query times are shown in Table 2.

**Table 2:** Total and per *k*-mer query times for the real (*P. aeruginosa*) and simulated (*Y. pestis*) dataset with peaks of main memory

	*P. aeruginosa*		*Y. pestis*	
	BFT	SBT	BFT	SBT
Total query time (min)	1.19	61.86	0.57	37.42
Query time per *k*-mer (μs)	7.14	371.16	3.42	224.52
Peak of main memory (MB)	2076	11,678	544	7775

A third experiment gives an estimation of the time required to traverse the graph represented by a BFT: It verifies for each *k*-mer of the batch queries whether its corresponding vertex in the graph is branching. This experiment first computes information about the root in a negligible amount of time and memory. Then, the BFT is queried for its branching vertices. For the real dataset, this experiment took 55.52 s (average time of 5.55 $\mu$s per 63-mer), resulting in 1,574,198 branching vertices. For the simulated dataset, this experiment took 38.79 s (average time of 3.88 $\mu$s per 54-mer), resulting in 141,802 branching vertices.

In summary, in our experiments the BFT was up to two times faster to build than the SBT while using about the same amount of main memory. When written on disk, the BFT used less memory than SBT for both datasets and when compressed with 7z, the BFT was about two times smaller than the SBT. For querying *k*-mers, the BFT was about 52 to 66 times faster than the SBT while using about 5.5 to 14.3 times less memory.

## Conclusions

We proposed a novel data structure called the Bloom Filter Trie for storing a pan-genome as a colored de Bruijn graph. The trie stores *k*-mers and their colors. A new representation of vertices is proposed to compress and index shared substrings. It uses four basic data structures that allow to quickly verify the presence of substrings. In the worst case, the compressed strings have a memory footprint close to their binary representation. However, we observe in practice substantial memory savings. Future work concerns the possiblity to compress non-branching paths that share the same colors [19] and also the extraction of the different pan-genome regions.
